# Calendar

**DOI:** 10.1155/S0962935192000152

**Published:** 1992


					Calendar
Calendar
1992
5-12 March, 1992
The American Academy of
Allergy & Immunology Annual
Meeting, Orlando, USA
Contact: The Executive Office,
AAAI, 611 East Wells Street,
Milwaukee, Wisconsin 53202,
USA
Tel: +1 414 272 6071
2-6 May, 1992
4th International Conference on
Tumor Necrosis Factor and
Related Cytokines
Veldhoven, The Netherlands
Contact: Secretariat, 4th
International TNF Congress,
Department of Surgery,
University of Limburg, PO Box
616, 6200 MD Maastricht, The
Netherlands
Fax: +31 43 437599
10-15 May, 1992
15th Congress of the European
Academy of Allergology and
Clinical
Immunology
Paris, France
Contact: Mrs J Siraudin, Congres
EAACI-Paris 1992, Clinique des
Maladies Respiratoires, Hopital
de l'Aiguelongue, Avenue du
Major Flandre, 34059 Montpellier
Cedex 1, France
Tel: +33 67 022020
Fax: +33 67 042000
26-30 May, 1992
First International Congress of
World Hungarian Medical
Academy (WHMA)
Balatonaliga, Hungary
Contact: Professor Sandor Szabo,
President of the Congress,
Department of Chemical
Pathology, Brigh and Woman's
Hospital, Boston, Massachusets,
USA
Tel: +1 617 732 5912
Fax: +1 617 732 4432
Symposium (at the above
congress): Mediators of
Inflammation
Contact: Professor IL Bonta,
Chairman, Erasmus University
Rotterdam
Tel: +31 10 4087546
Fax: +31 10 4360364
25-27 June, 1992
2nd Frankfurt International
Cytokine Symposium
Frankfurt, Germany
Contact: Priv.-Doz. Dr. L.
Bergmann, Mrs A Hipfel,
Division of Haematology,
Department of Internal Medicine,
University of Frankfurt, Theodor-
Stern-Kai 7, D-6000 Frankfurt,
Germany
Tel: +49 69 6301-5744
Fax: +49 69 6301 6301
26-31 July, 1992
8th International Conference on
Prostaglandins
Montreal, Canada
Contact: Professor M
Rola-Pleszczynski, MD, Head of
Immunology Division, Faculty of
Medicine, University of
Sherbrooke, 3001, 12th Avenue
North, Sherbrooke, OC, J1H 5N4,
Canada
Tel: +1 819 5645211
Fax: +1 819 5645378
23-28 August, 1992
8th International Congress on
Immunology
Budapest, Hungary
Contact: Professor J Gergely,
President of the Congress,
Intercongress, H-1068 Budapest,
Hungary
Dozsa Gy. ut 84/a
Tel; +36 1 1424118
Fax: +36 1 1428130
1993
12-17 March, 1993
The American Academy of
Allergy & Immunology Annual
Meeting,
Chicago, USA
Contact: The Executive Office,
AAAI, 611 East Wells Street,
Milwaukee, Wisconsin 53202,
USA
Tel: +1 414 272 6071
Rapid Communications of Oxford Ltd Mediators of Inflammation. Vol 1992 75

				

